# Supplementary figures and images for: An autoimmune disease risk variant: A trans master regulatory effect mediated by IRF1 under immune stimulation?
Source: PLoS Genet. 2021 Jul 27;17(7):e1009684. doi: 10.1371/journal.pgen.1009684 (PMC8345867; doi:10.1371/journal.pgen.1009684)

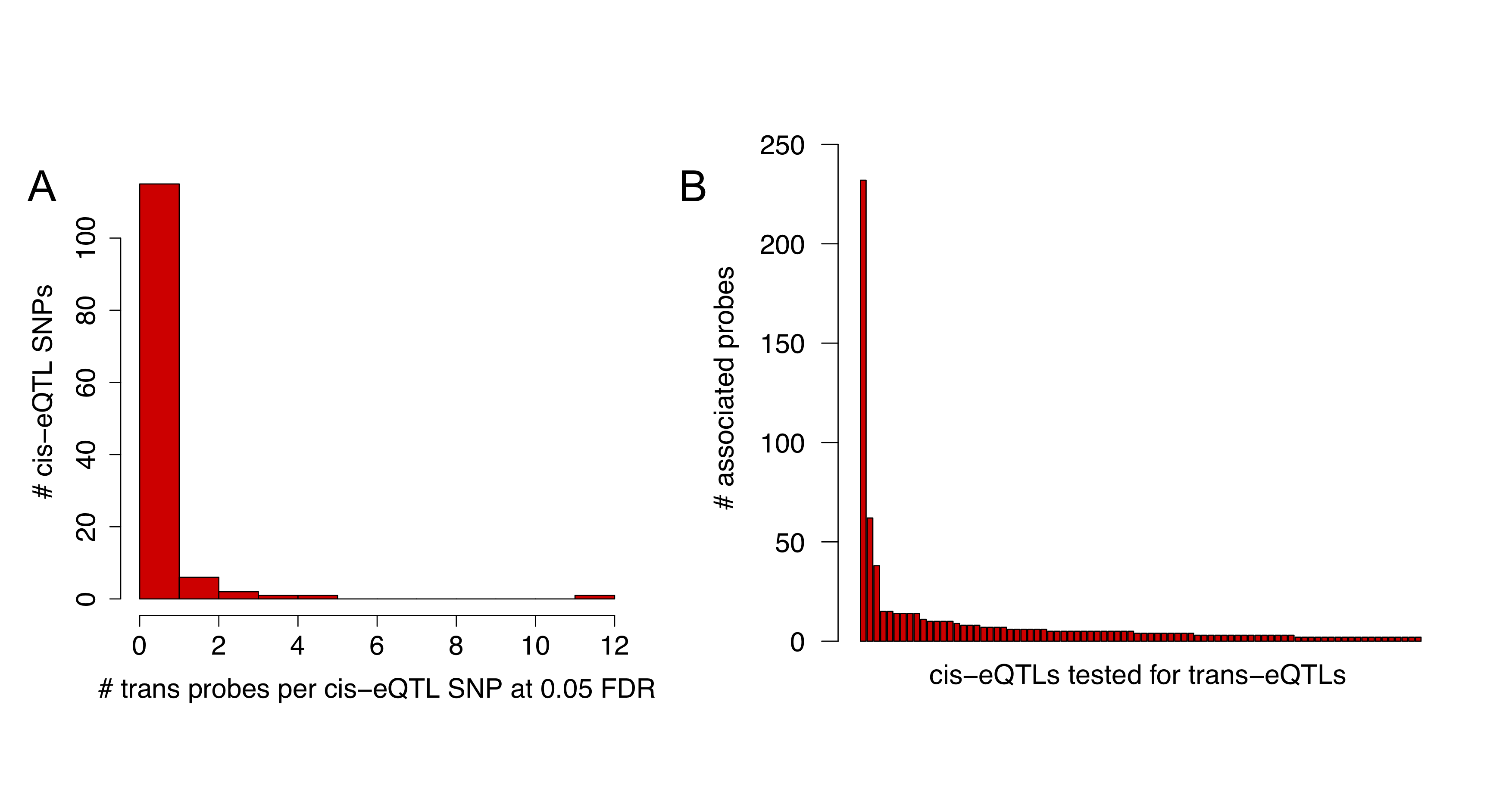

Supplement: S1 Fig — Histogram of the number of trans probes associated to each cis-eQTL SNP in monocyte trans-eQTL study at 0.05 FDR (A) and number of probes associated with each SNP at 0.5 FDR (B). (TIFF) [file pgen.1009684.s008.tiff]

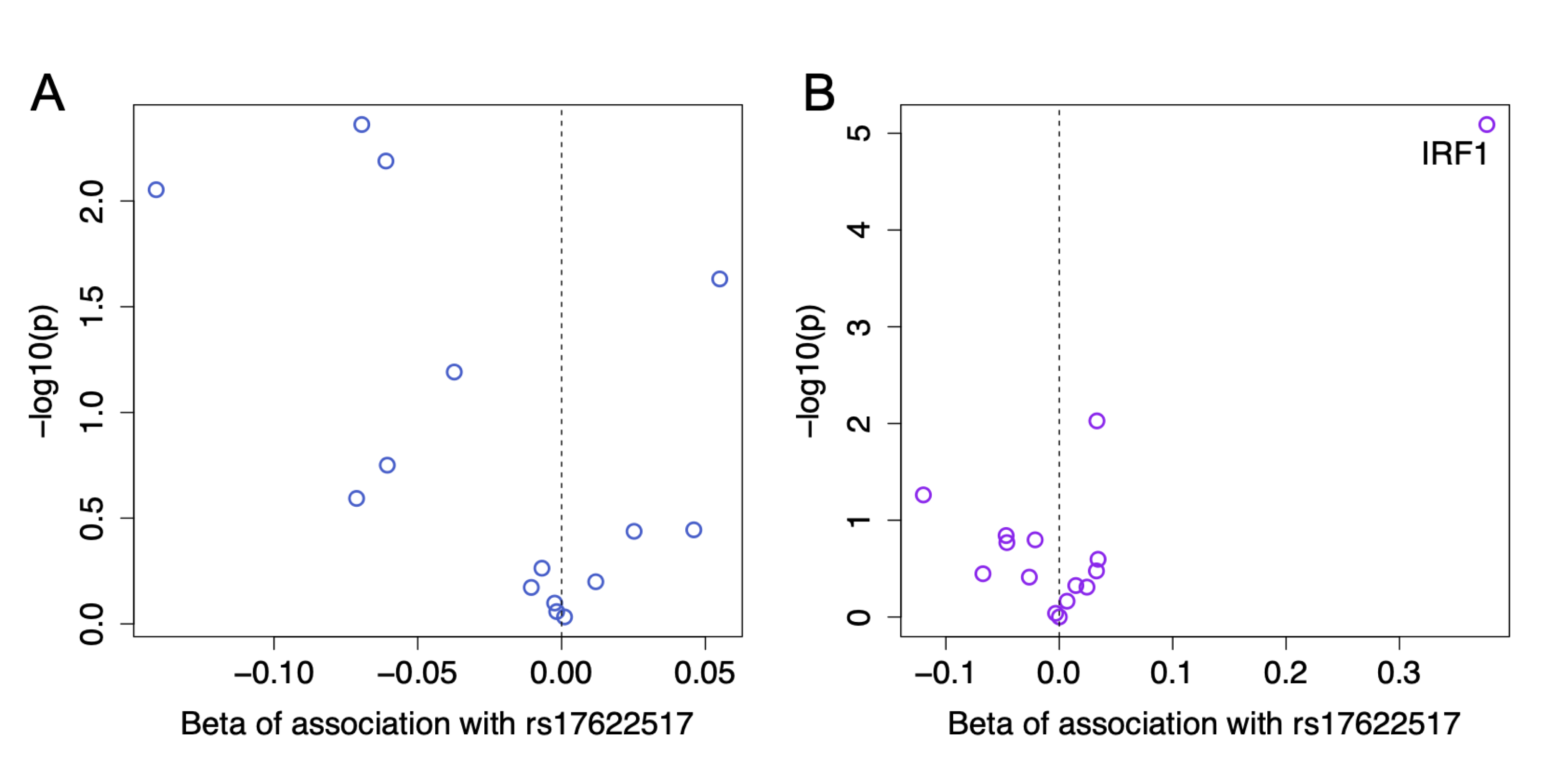

Supplement: S2 Fig — rs17622517 cis association with expression of genes within 1 MB in monocytes in control (A) and LPS 90 min stimulation (B). (TIFF) [file pgen.1009684.s009.tiff]

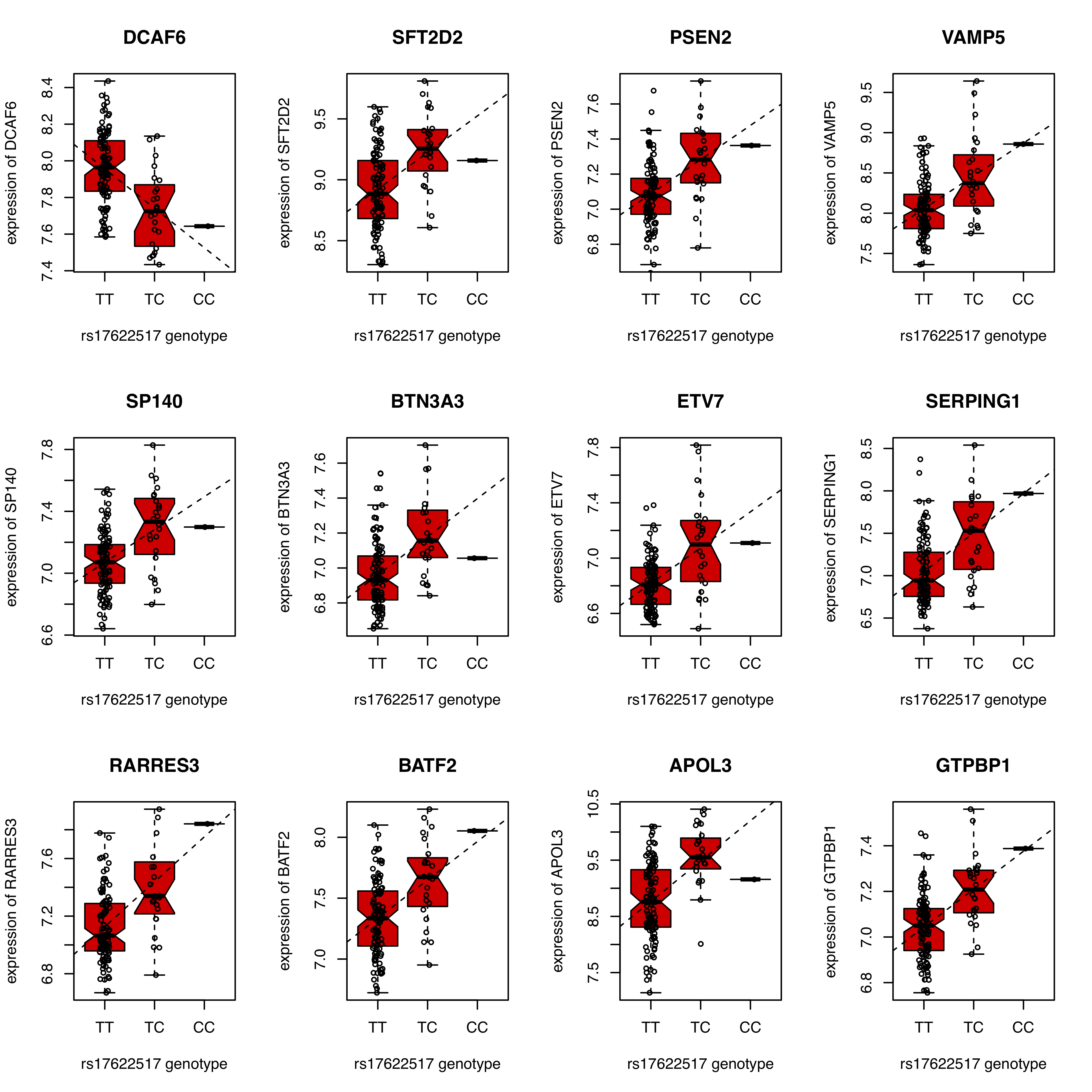

Supplement: S3 Fig — Twelve most significant trans-eQTLs associated with rs17622517 under 6h LPS at FDR < 0.05. (TIFF) [file pgen.1009684.s010.tiff]

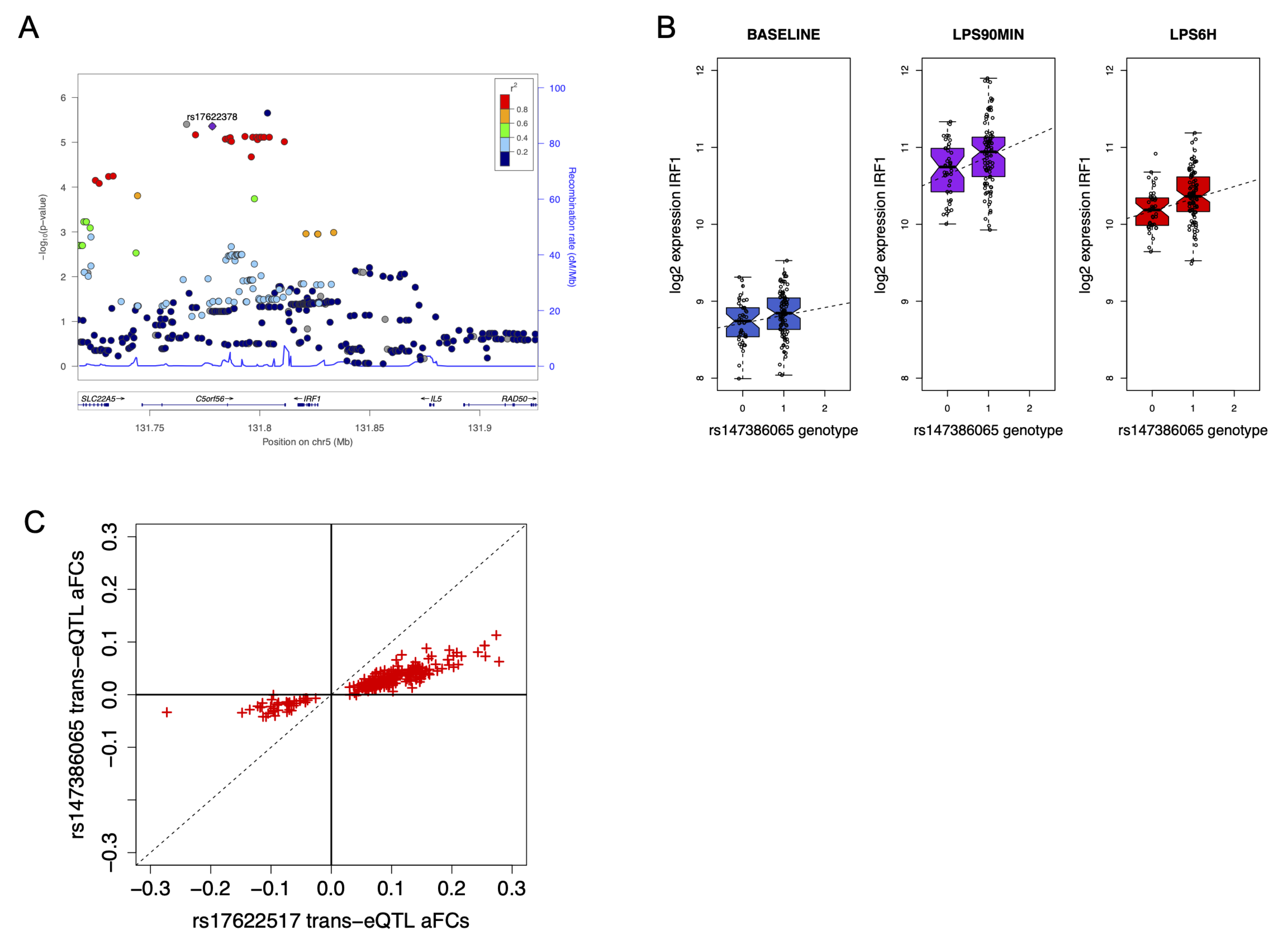

Supplement: S4 Fig — A) Locus zoom plot highlighting LD pattern for second top SNP in IRF1 cis-eQTL and B) cis-eQTL effect of rs147386065 on IRF1, C) trans-eQTL effect sizes of the significant rs17622517 trans-eQTLs (FDR < 50%) versus effect sizes of rs147386065 trans effects on the same genes. (TIFF) [file pgen.1009684.s011.tiff]

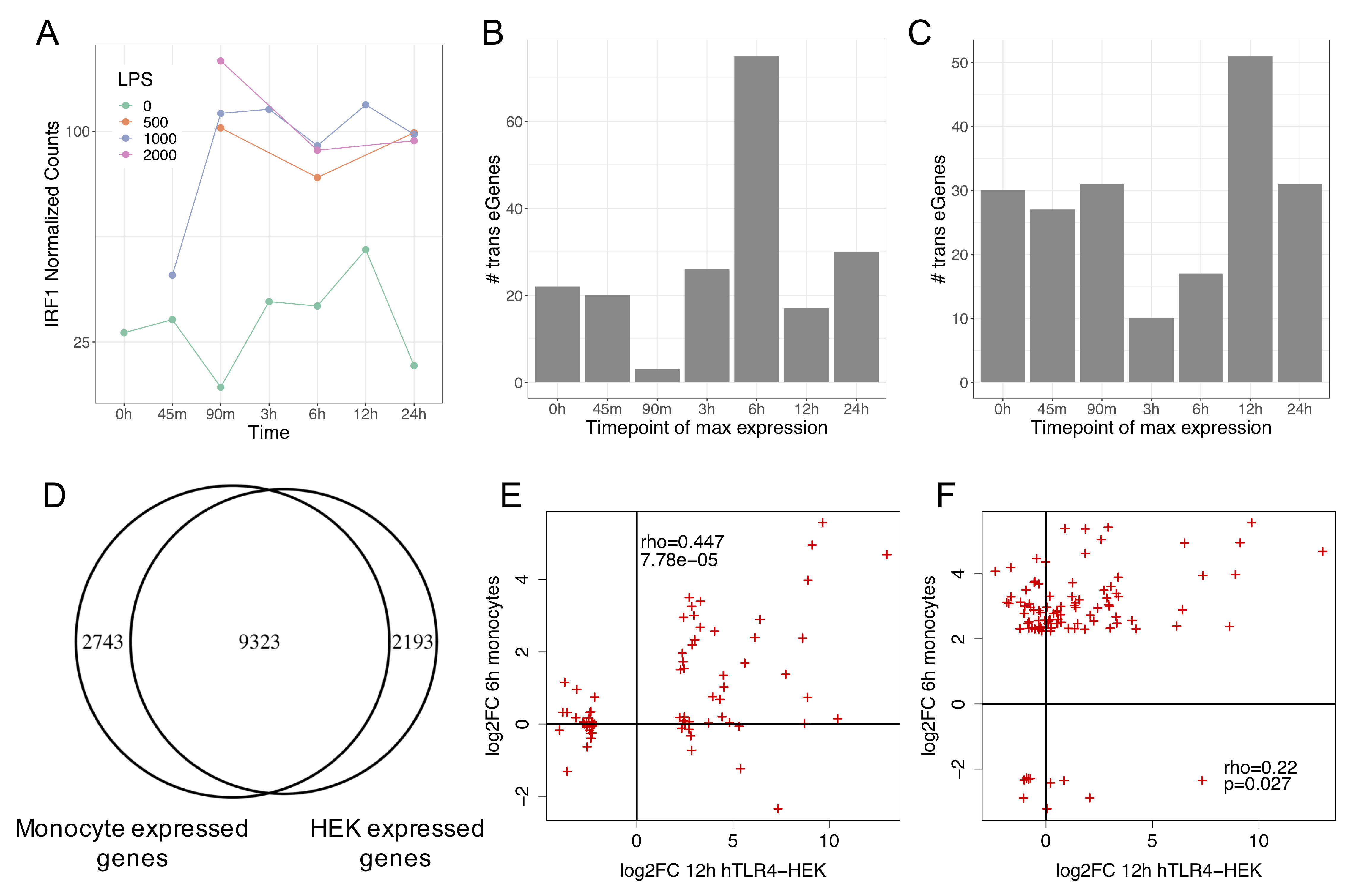

Supplement: S5 Fig — A) expression of IRF1 for the different conditions; B-C) number of rs17622517 trans-eGenes that have their maximum expression at each time point in primary monocytes using data from Kim-Hellmuth et al. 2017 (B), and in HEK293-TLR4 cells (C). The peak trans-eGene expression is at 6 hours in monocytes and at 12 hours in HEK293-TLR4 cells. D) Overlap of genes expressed in monocytes and HEK-hTLR4 cells. Monocyte expression was quantified with microarray (in Kim-Hellmuth, 2017) where expressed probes were defined as having a p value of detection less than 0.0001. HEK293-hTLR4 cells were quantified with RNA-seq and expressed genes were defined as having an average expression across samples of at least 5 reads. E) Correlation of the log2 fold change (log2FC) in HEK-hTLR4 versus monocytes for the top 100 differentially expressed genes (by absolute value of fold change) in HEK-hTLR4 cells. F) Correlation of log2FC in HEK-hTLR4 versus monocytes for the top 100 differentially expressed genes in monocytes. (TIFF) [file pgen.1009684.s012.tiff]

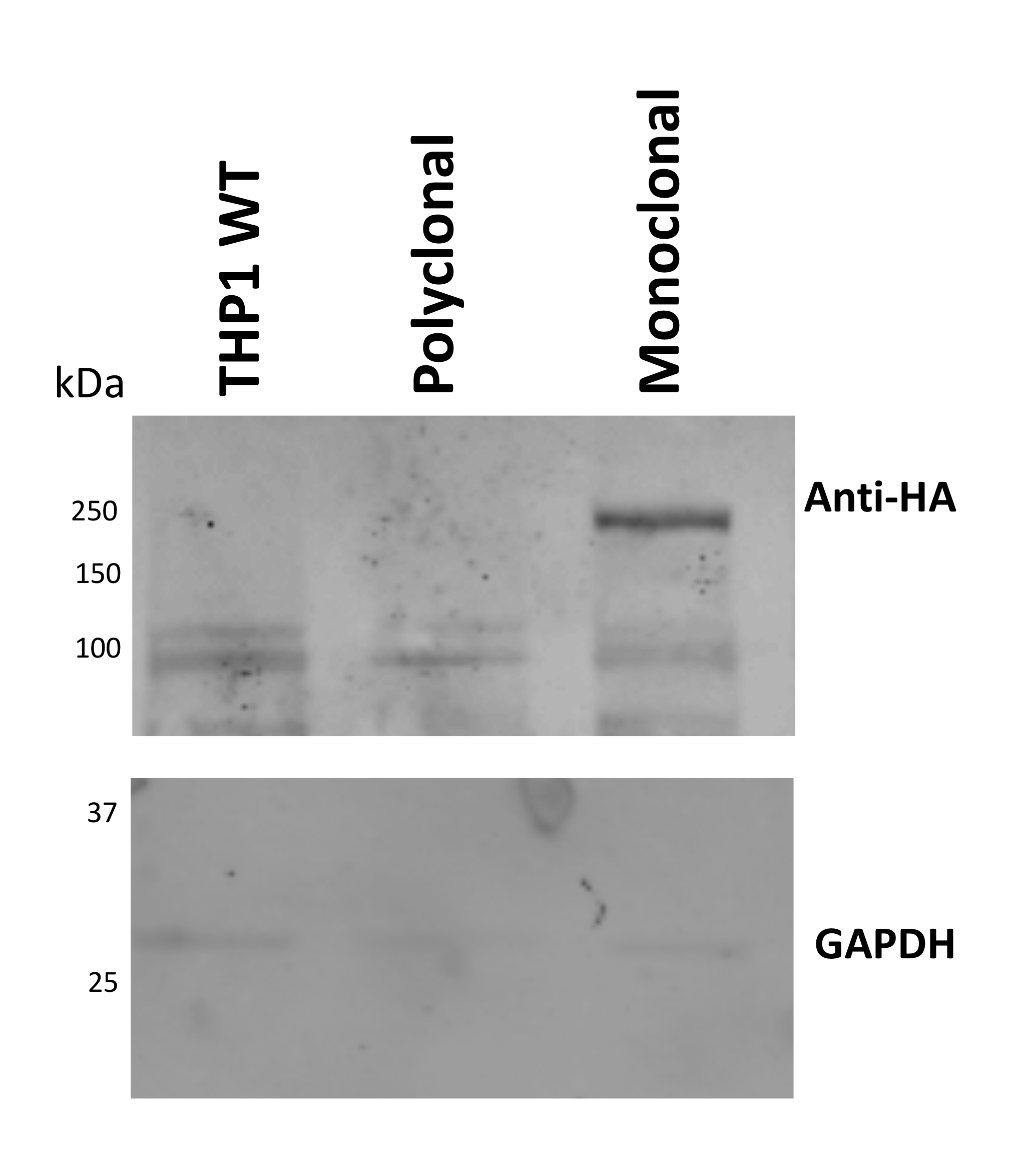

Supplement: S6 Fig — Western blot with anti HA-tag of THP1 WT, THP1 KRAB-dCas9-MeCP2 polyclonal and monoclonal cell lines. Mouse GAPDH was used as loading control. (TIFF) [file pgen.1009684.s013.tiff]

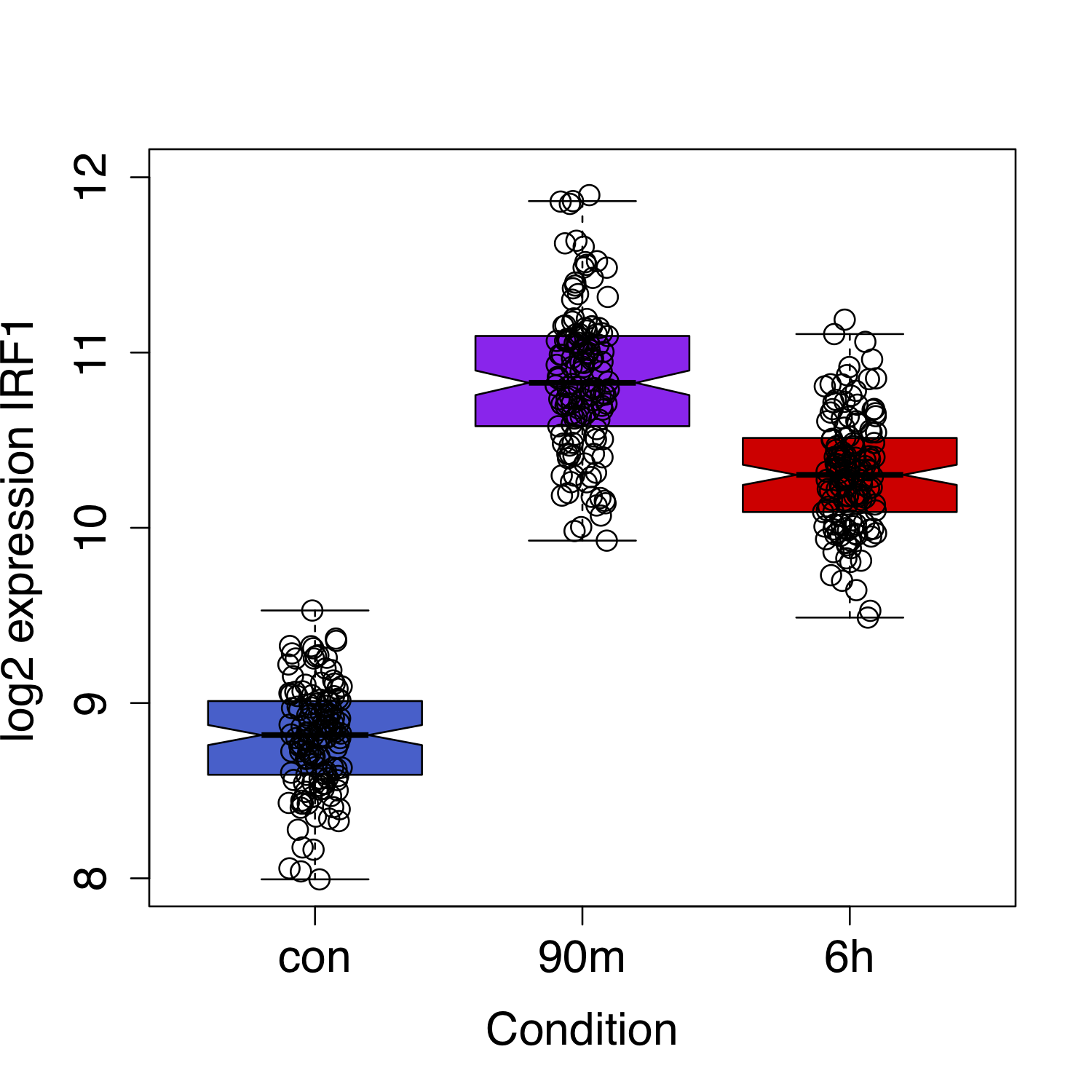

Supplement: S7 Fig — Expression change in IRF1 for 134 donors from Kim-Hellmuth, 2017), showing a mean fold change of 1.23 between control and LPS 90m samples (Wilcoxon p < 2.2−16). (TIFF) [file pgen.1009684.s014.tiff]

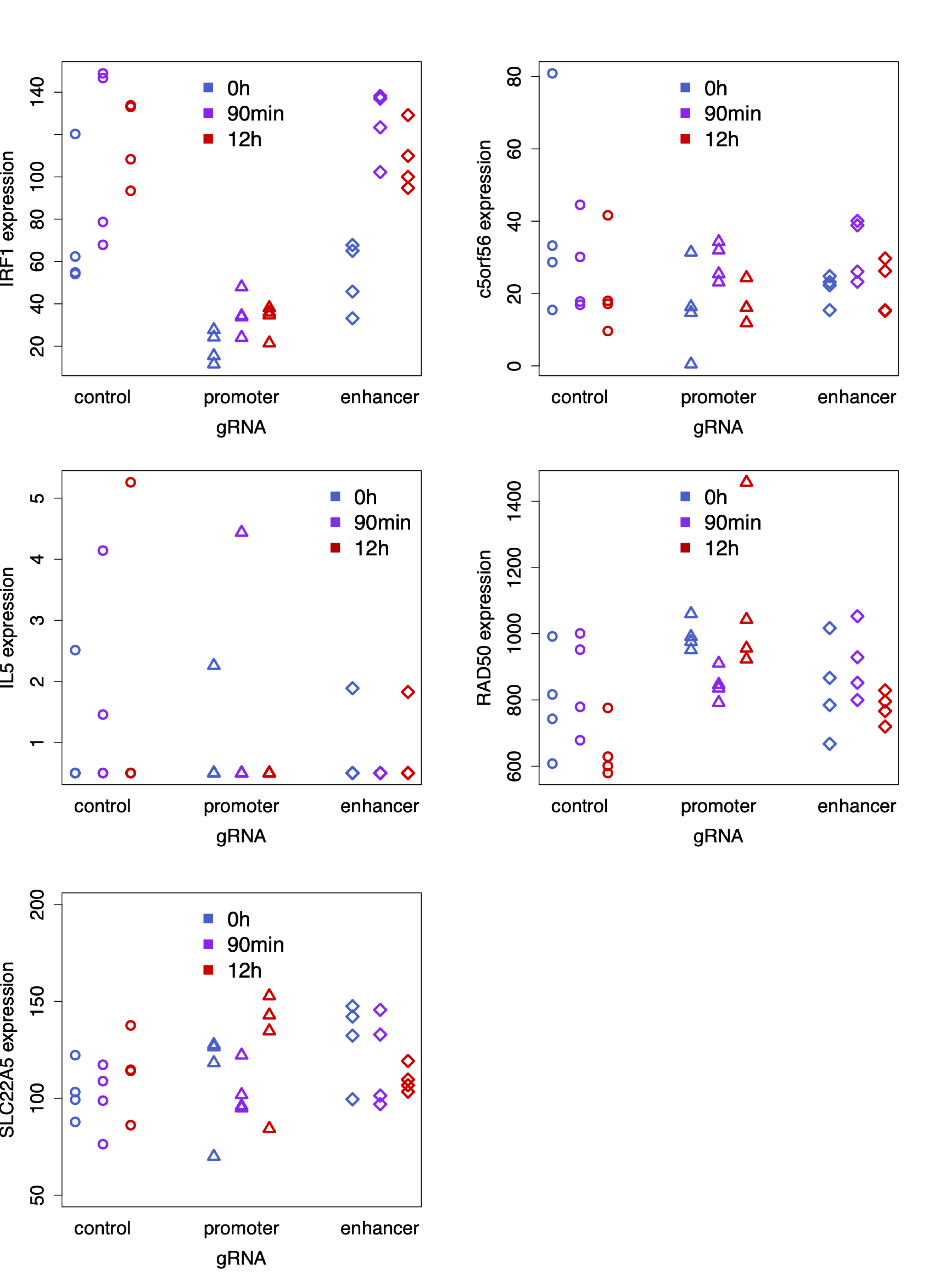

Supplement: S8 Fig — Expression levels of the genes within 1Mb in HEK293-TLR4 cells with and without CRISPRi silencing of the rs17622517 locus. The lack of significant difference indicates that this putative enhancer does not have a strong effect on other genes in cis. (TIFF) [file pgen.1009684.s015.tiff]

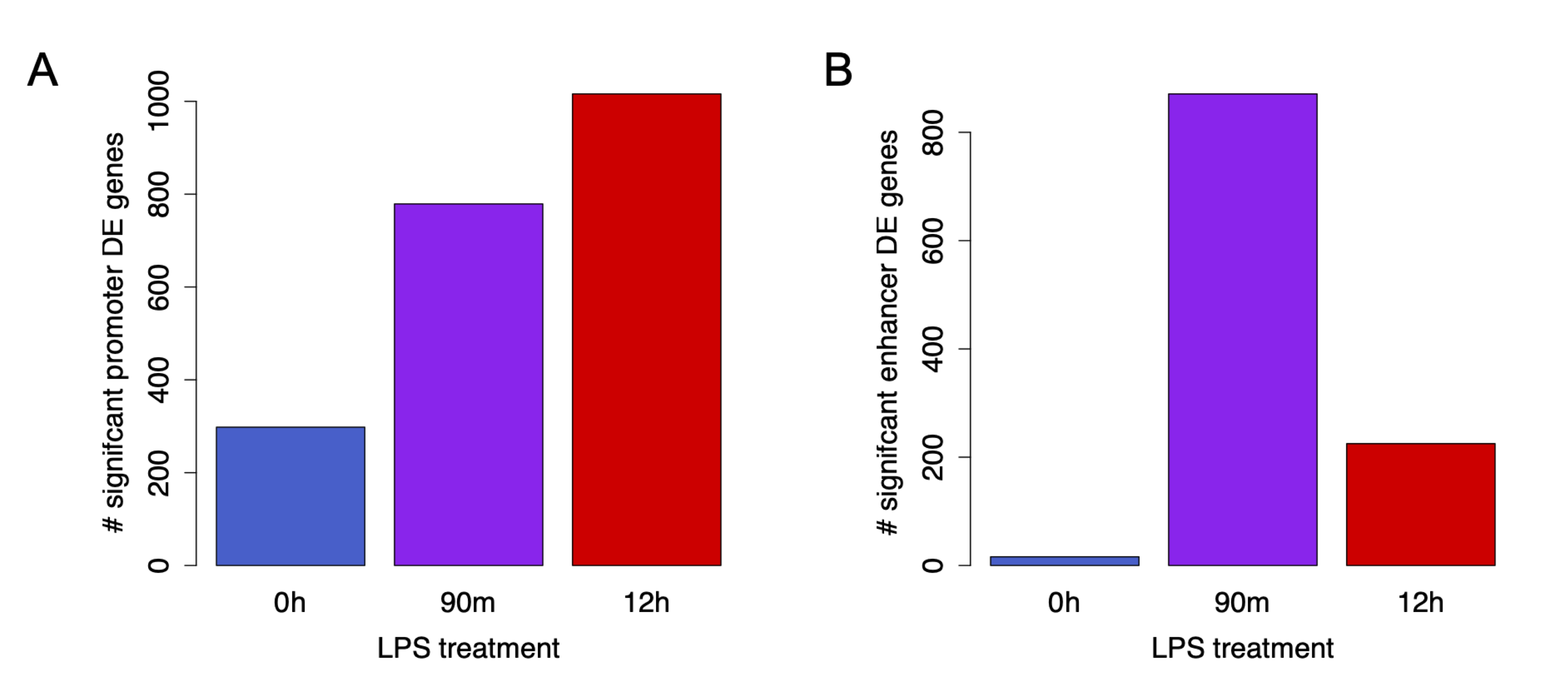

Supplement: S9 Fig — Number of significantly differentially expressed genes in promoter (A) and enhancer (B) versus controls at the respective LPS condition. (TIFF) [file pgen.1009684.s016.tiff]

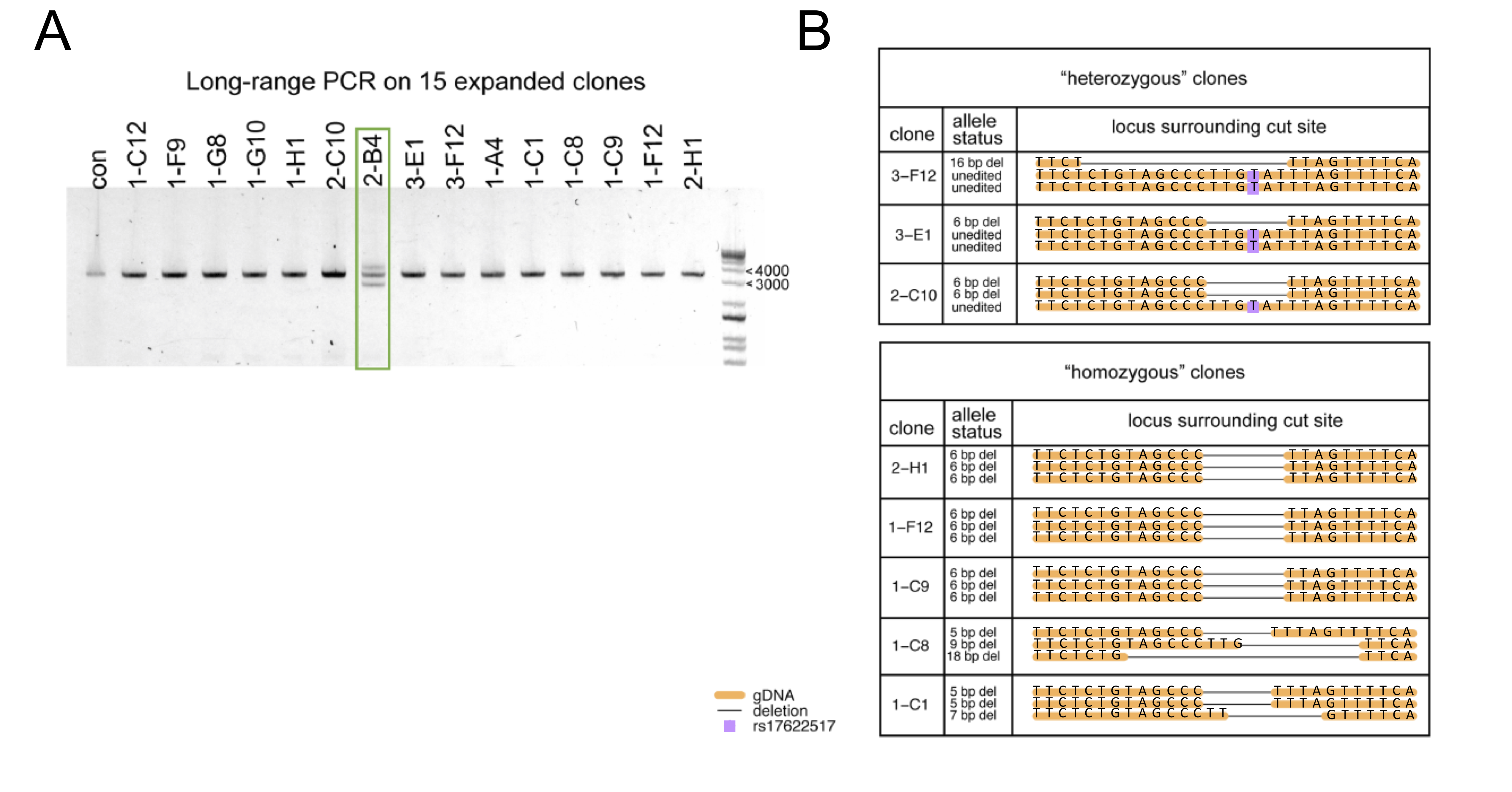

Supplement: S10 Fig — A) Electrophoresis of long-range PCR products showed a large deletion in one clone (green) with that was excluded from further analysis; B) genotypes of clones selected for analysis. (TIFF) [file pgen.1009684.s017.tiff]

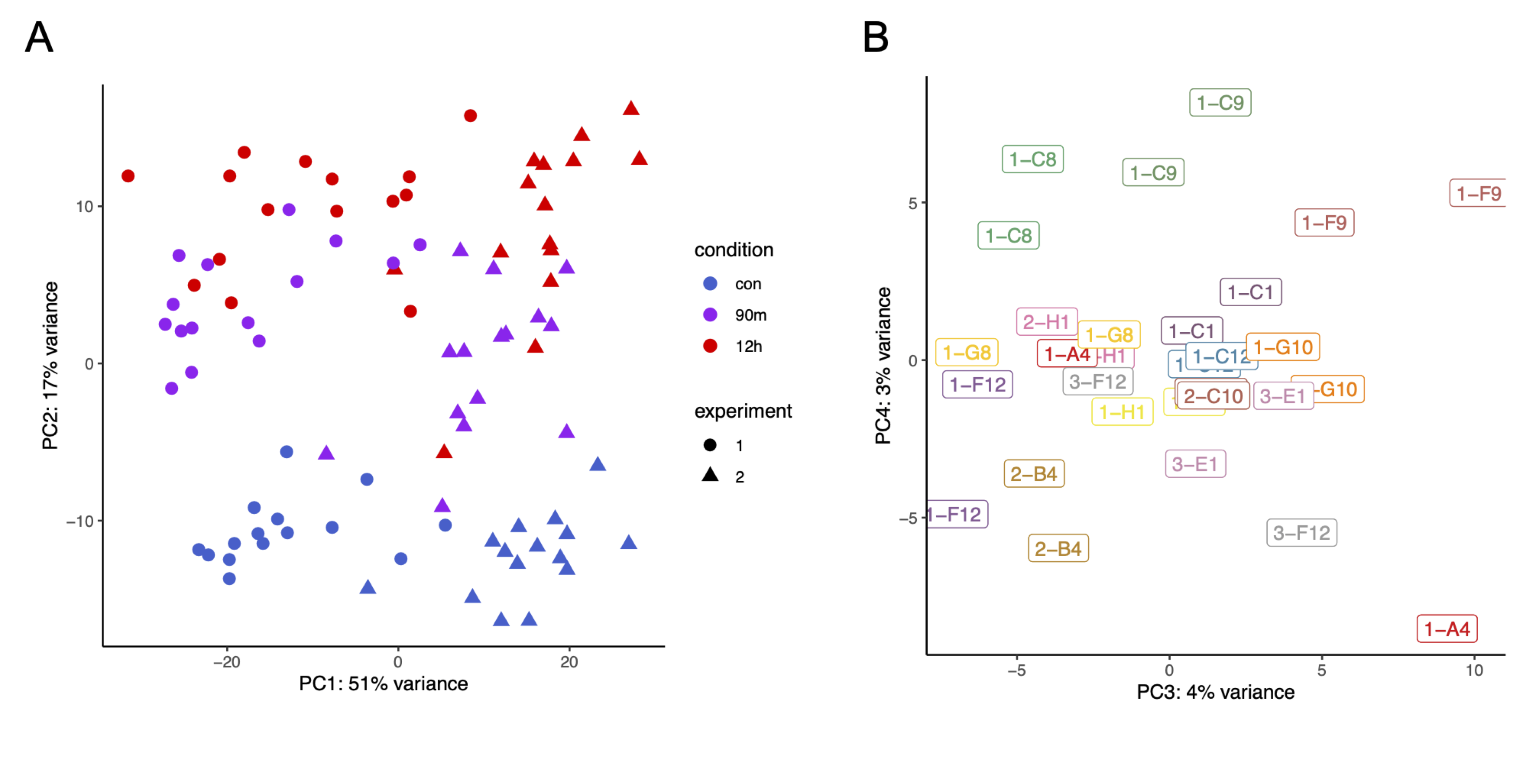

Supplement: S11 Fig — A) Principal components 1 and 2 for all clones across both experiments, highlighting condition and experiment. B) Principal components 3 and 4 for all clones under control conditions, highlighting clone identity. (TIFF) [file pgen.1009684.s018.tiff]

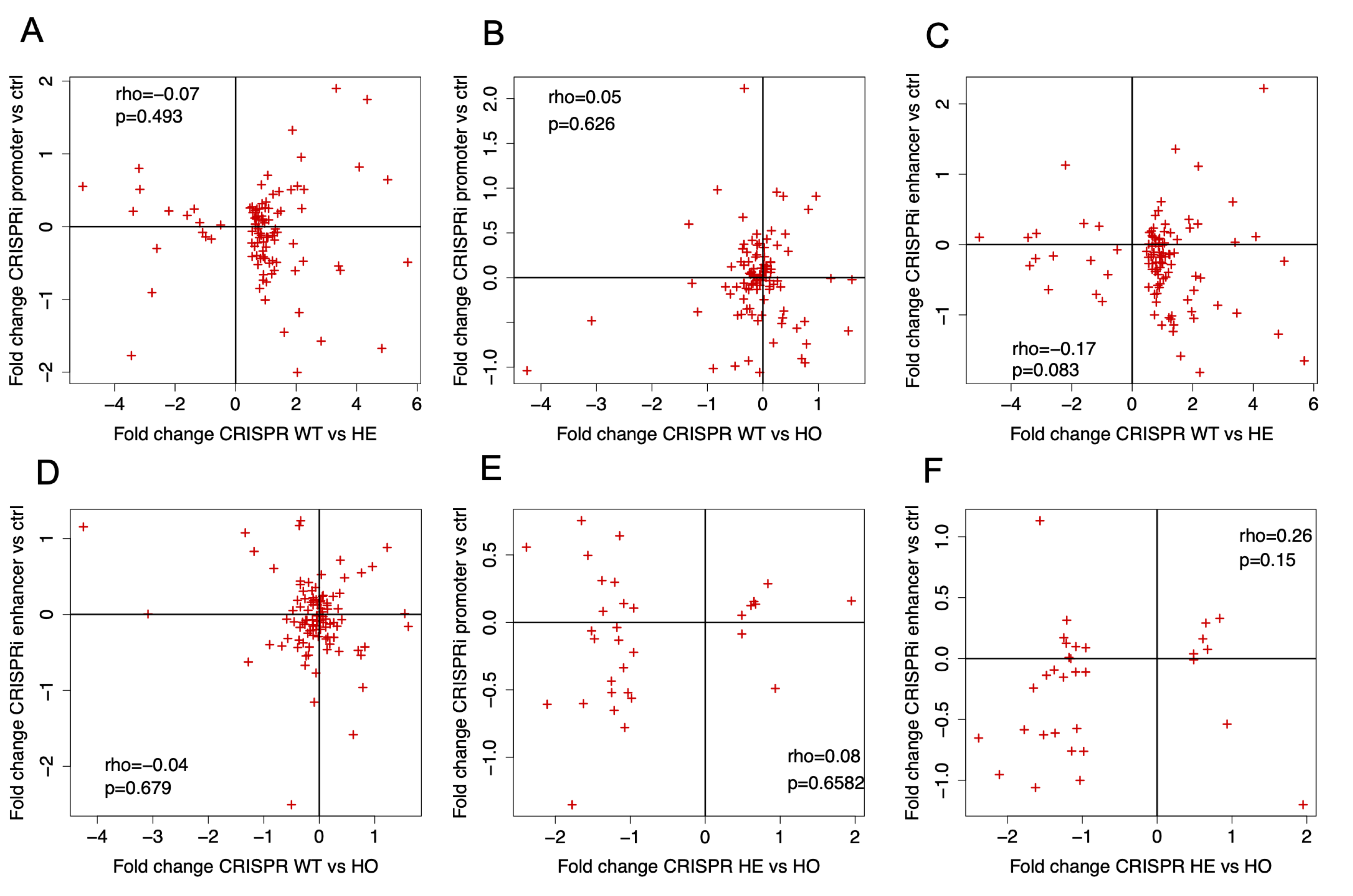

Supplement: S12 Fig — Correlation of gene expression fold changes at 12h LPS condition for the top 100 differentially expressed genes (by fold change) in wild type versus heterozygous clones (A & C) and wild type versus homozygous clones (B & D) versus fold change for promoter (A & B) and enhancer (C & D) CRISPRi silencing. Correlation of 0.05 FDR differentially expressed genes in heterozygous versus homozygous clones versus fold change for promoter (E) and enhancer (F) CRISPRi silencing. (TIFF) [file pgen.1009684.s019.tiff]

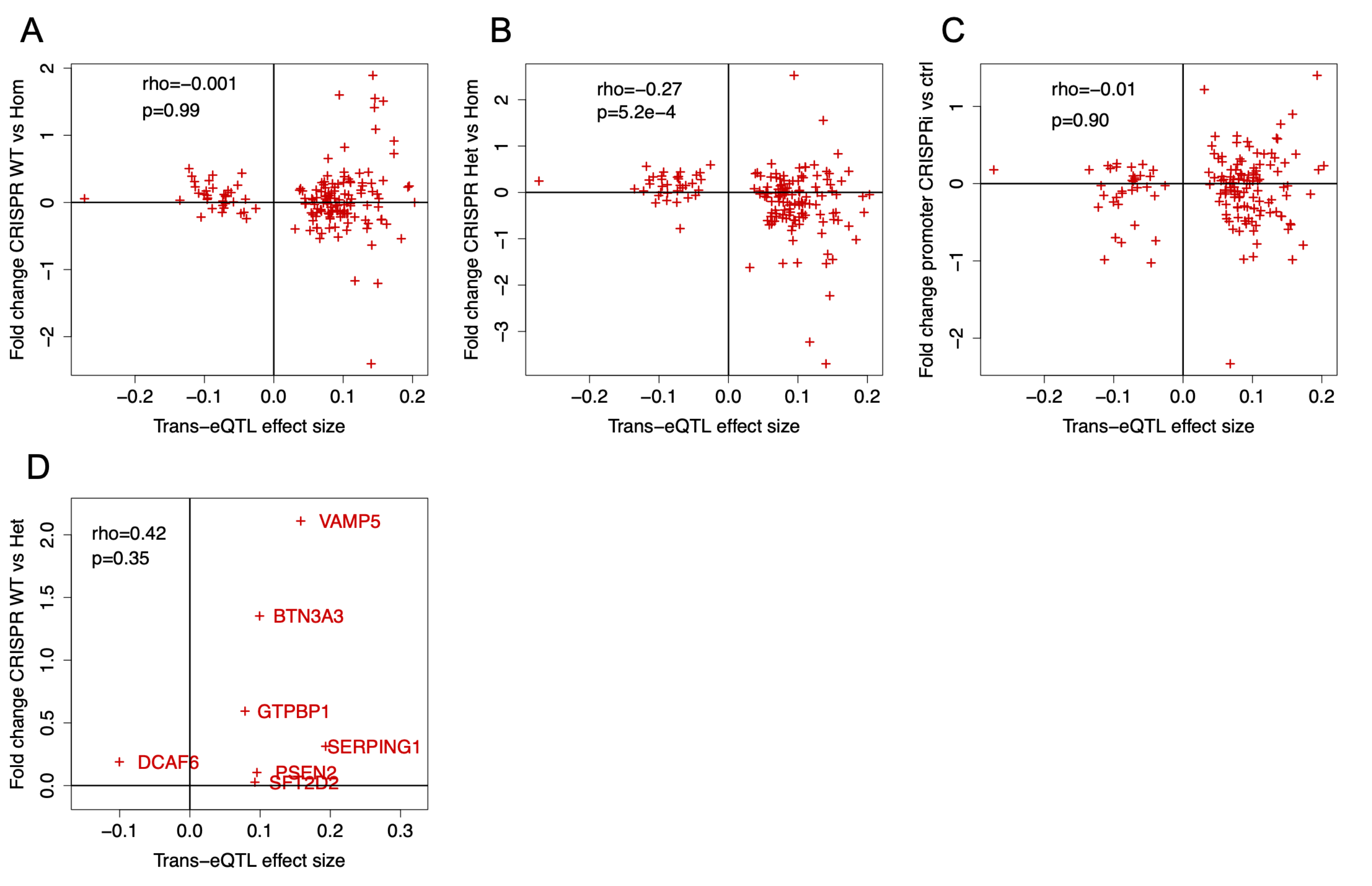

Supplement: S13 Fig — Correlation of 0.5 FDR trans-eQTL effect size (allelic fold change) with differential expression in (A) wild-type versus CRISPR-edited homozygous clones, (B) CRISPR-edited heterozygous versus homozygous clones and (C) CRISPRi promoter silencing versus control samples. D) Correlation of 0.05 FDR trans-eQTL effect size with differential expression in CRISPR edited heterozygous clones. (TIFF) [file pgen.1009684.s020.tiff]
