# Supplementary material for: An autoimmune disease risk variant: A trans master regulatory effect mediated by IRF1 under immune stimulation?
Source: PLoS Genet. 2021 Jul 27;17(7):e1009684. doi: 10.1371/journal.pgen.1009684 (PMC8345867; doi:10.1371/journal.pgen.1009684)
Supplement: Attachment — Submitted filename: response to reviewers.pdf [file pgen.1009684.s021.pdf]

Response to reviewers

Reviewer #1:

The authors map trans eQTLs in monocytes from 134 donors stimulated with LPS for 6h, restricting the analysis to SNPs previously identified as response cis-eQTLs at an earlier timepoint (90 mins LPS treatment). They identify a locus that is a cis-eQTL for IRF1 and is associated with a large number of IRF1 target genes in trans, as well as being a GWAS locus. They aim to validate this putative enhancer using CRISPRi and CRISPR in a modified HEK293 cell line, and present a model of how the risk allele impacts gene expression.

It is important to conduct these functional analyses to follow up eQTL studies and I appreciate the challenges with being able to design the perfect experiment to directly address the difficult problem of identifying causal SNPs and genes. However, my major concern is that the results do not support the strong conclusions made in the manuscript. The results presented are only suggestive of a role of IRF1 and rs17622517 in the nearby enhancer locus and additional work will be required to confirm they are the causal gene and SNP.

RE: We appreciate the reviewer's concerns. As detailed below, we have addressed them in revisions by performing additional experiments and analysis that support the mediating role of IRF1 and this SNP as a functional variant. Since the term "causal variant" can be problematic despite its widespread use, we now predominantly refer to this SNP locus being functional, which we have direct evidence of.

Major comments:

1. The results of the CRISPRi experiment do not support the strong conclusion that IRF1 is downregulated by the enhancer silencing

a. As stated on page 10 "The effect of the enhancer silencing is not significantly different from the controls for IRF1". Given that downregulation of IRF1 was not observed I would suggest that the later sentence "The differential expression of promoter and enhancer have a strong positive correlation...which indicates that enhancer silencing downregulates IRF1" is too strong a conclusion and should be rephrased.

b. On page 10: "the response being similar to the IRF1 promoter silencing further provides strong evidence that it is indeed an enhancer of IRF1". This evidence is suggestive rather than strong.

RE: We have performed additional experiments to address this concern and also revised the text. We performed CRISPRi silencing of the promoter and enhancer in the THP1 monocyte-like cell line, and showed that the enhancer silencing indeed affects IRF1 expression with upregulation without LPS stimulus and suggestive downregulation at 90 mins. These results are included in Fig 2b and shown here. This result provides bona fide evidence that the locus is an enhancer of *IRF1*.

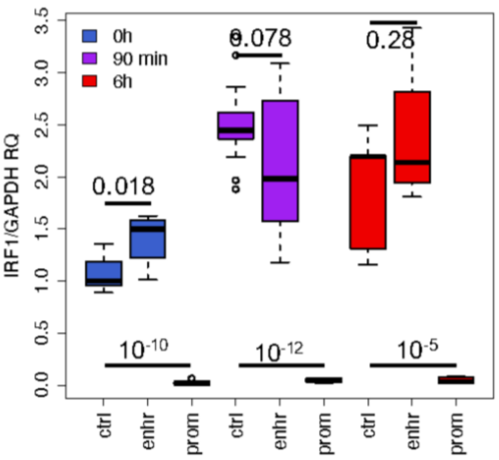

Furthermore, we now show that this variant is not a cis-eQTL for any other gene in our monocyte data (Supplementary Figure 2, copied below). We highlight that our claim of causality of IRF1 is not reliant on the CRISPR experiments alone; the cis/trans-eQTL discovery is also an important line of evidence.

Altogether, we feel that our combined eQTL and experimental results strongly support the mediating role of IRF1. There are multiple lines of evidence: 1) the two independent cis-eQTL effects on IRF1 (and no other genes in cis) with concordant trans-eQTL signals, 2) differential expression of IRF1 after silencing the enhancer locus in THP1 cells, 3) similar genome-wide expression effect of enhancer and promoter silencing as well as genomic perturbation of the locus.

However, we agree that the original wording in reference to the original CRISPRi results was too strong and have rephrased the quoted sentences to:

"The differential expression of promoter and enhancer have a strong positive correlation (rho = 0.89, p<2.2e-16, Fig. 2d), which suggests that the impact of enhancer may be mediated by downregulation of IRF1.", and *"Together with the qRT-PCR results showing that this enhancer affects IRF1 and the transcriptome response being similar to the IRF1 promoter silencing, our results indicate that this locus is an enhancer of IRF1."*

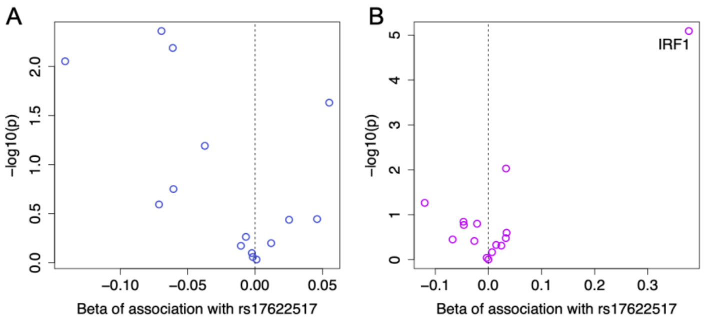

c. Figure 2c: Is the upregulation of IRF1 expression in response to LPS less than would be expected based on the previous microarray study and the response in the CRISPR experiment? While statistically significant, from the figure it looks like a very modest upregulation in expression.

RE: To address this question, we looked at IRF1 expression in the primary monocytes from the 134 original study participants (Kim-Hellmuth et al, 2017). Plotted below is the effect of LPS treatment on IRF1 expression in these samples. While there is an effect of LPS on IRF1 in monocytes (Wilcoxon *p<2.2e-16*, 90m versus 0h), the effect size is moderate (1.23 average fold change between 90m and 0h). In this CRISPR experiment in HEK-TLR4 cells, we see an average fold change of 1.75 between 90m and 0h in the control samples. In summary, the upregulation to LPS is not less than what we would expect. We have now added this in the manuscript text as: "*The fold change of 1.75 is comparable to that observed in the original primary monocyte data (1.23, Wilcoxon p = $p<2.2^{-16}$, Supplementary Figure 7).*"

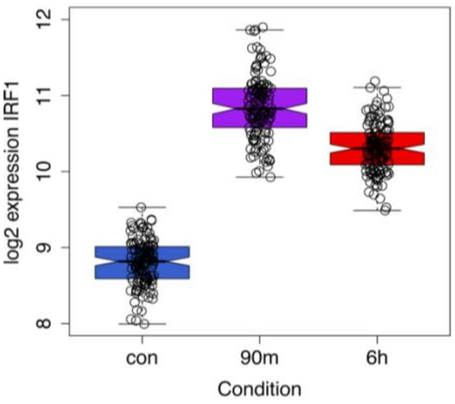

d. On page 14 the authors state that "CRISPRi is suboptimal for recapitulating eQTL effects driven by genetic variants". Could they expand on the motivation for using this technique for this eQTL validation experiment? Have the authors considered if any other experiments could be more informative for determining if the rs17622517 locus is an enhancer for IRF1 e.g. chromatin conformation capture?

RE: Our motivation for using CRISPRi was that it is an adaptable and established protocol for linking enhancer regions to genes. This statement on page 14 was meant to convey the limitation of methods such as CRISPRi (as well as chromatin capture assays), which might be effective for linking enhancers to promoters, but may be more limited to recapitulate the specific effect of a genetic variant than CRISPR editing. We have clarified it as follows:

"CRISPRi is suboptimal for recapitulating eQTL effects driven by genetic variants while being very powerful for demonstrating enhancer function."

e. As described in the points above, the following sentence in the abstract referring to the function as an IRF1 enhancer feels too strong given the results of the CRISPRi silencing experiment "Using CRISPRi silencing, we showed that the SNP locus indeed functions as an IRF1 enhancer with widespread transcriptional effects."

RE: Given the additional experimental support for this locus being an enhancer for IRF1, and a fair point about the needing to soften the statement, we adjusted accordingly this sentence in the abstract:

"Using CRISPRi silencing, we showed that perturbation of the SNP locus downregulates IRF1 and causes widespread transcriptional effects. Genome editing by CRISPR had suggestive recapitulation of the LPS-specific trans-eQTL signal and lent support for the rs17622517 locus being causal. "

2. On page 10, could the authors expand upon why they hypothesise that they are "underpowered to detect an effect on IRF1 in cis, but might capture its trans effects" given there is usually better power for the detection of cis effects?

RE: While we agree that *cis* effects tend to be larger and easier to detect and that the individual trans effects are weak and noisy, our rationale in this point is that looking at a lot of genes compensates for the noise in any individual gene. To clarify in the text, we rephrased the statement to:

"...we hypothesized that we may be underpowered to detect an effect on IRF1 in cis, but might capture its trans effects by counteracting the noise present in individuals genes by looking at many genes at once."

3. The results of the CRISPR experiment do not support the conclusion that rs17622517 is the causal SNP driving the cis and trans eQTL effects. The experiment conducted provides additional supportive evidence for the role of that locus in the regulation of IRF1 expression, but it still doesn't address the hypothesis that rs17622517 is the causal SNP.

a. Could the authors elaborate on what the correct interpretation of NHEJ is in the context of an eQTL? How should the reader interpret the deletion of the eQTL allele rather than a substitution?

RE: We had originally aimed to edit the exact variant with homologous recombination, but homologous recombination efficiency was so low for this locus that it was unfeasible to obtain a sufficient number of clones. Therefore, we pursued NHEJ as a way to measure the effect of perturbation of the genetic locus with replicates of each genotype. We agree that this does not necessarily recapitulate the effect of the variant, but our results clearly indicate that this specific locus is functional rather than a nonfunctional LD proxy of another causal variant. We have expanded on this in the discussion:

"While editing the exact variant into a cell line may reflect directionally the same genetic effect as seen in the population, a deletion of a locus could feasibly have an effect in the same direction, opposite direction or not at all. This depends upon the specific mechanism of the genetic effect. For example, if a variant increases the affinity of a DNA binding protein by modifying its binding motif, that effect may not be replicated by a simple deletion of the variant locus. However, a functional impact

of indels does provide evidence that the specific SNP locus has a regulatory effect rather than being a nonfunctional LD proxy of another causal variant. "

b. On page 12 it is very hard to see the "potential slight genotype effect" particularly in fig 3a.

RE: We agree that this effect is too subtle to mention in the text, so we deleted that part of the sentence.

c. Although the correlations between CRISPR and CRISPRi are statistically significant, they are very weak and what looks like nearly half of the genes show opposite effects between the two experiments.

RE: We selected the gene set for this analysis using an FDR cutoff of 0.25, which means roughly 25% of the genes are expected to be false positives and are expected to contribute to the noise in the correlation. We reran this analysis using a 5% FDR, and the enhancer shows a similar correlation, although it is not significant due to the low number of data points. We have included the plots at FDR 0.05 in Supplementary Figure 12, and below. We have also softened the language concerning the results and interpretation of the CRISPR experiment.

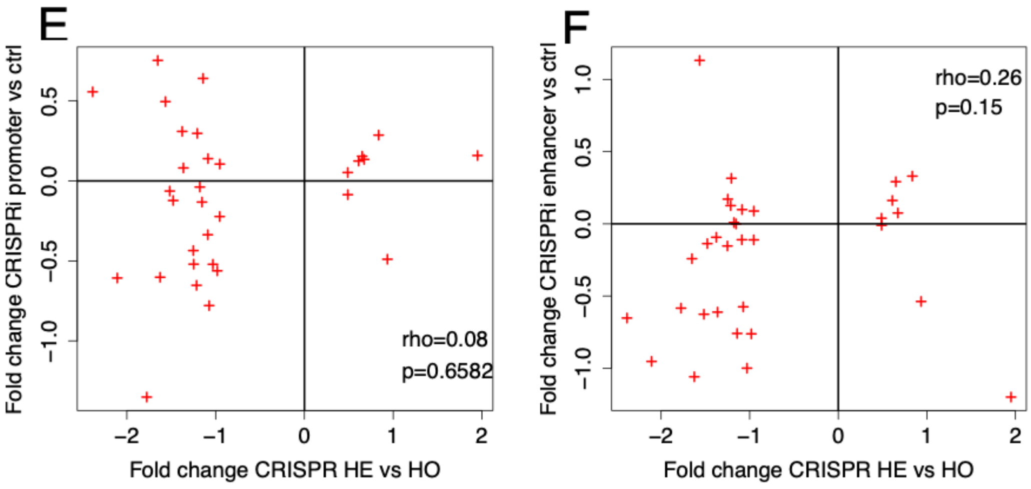

d. As described in the points above, I do not agree that the results presented support the sentence on page 13 "Having provided experimental evidence that rs17622517 is the causal eQTL variant"

RE: We agree that this phrasing is too strong and have rephrased the beginning of the sentence to "Having suggestive experimental evidence...".

e. Fig 4a shows a weak correlation between the trans-eQTL effect size and the CRISPR experiment which is being driven by a small number of genes. Given that such a relaxed FDR threshold of 0.5 has been used, could the authors comment on whether the genes

that do show a good correlation between the experiments are those with the more significant trans eQTL FDR?

RE: The correlation is indeed weak, but one must consider that we are comparing a genetic association in primary cells to effects of mixed indels in a cell line. These are not replication experiments but highly orthogonal validation experiments with many biological sources of variance. We also plotted the same correlation with a 0.05 FDR threshold and find that they have a strong correlation, albeit not significant due to low number of genes (new Supplementary figure 13d, right).

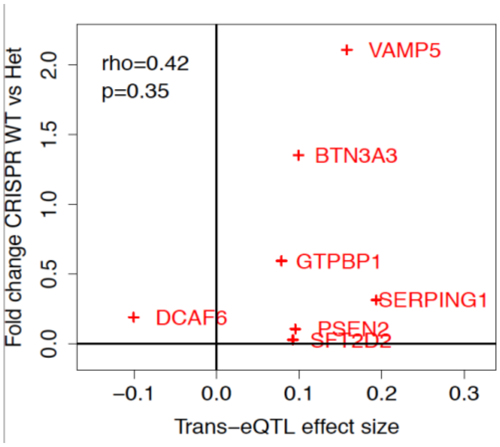

f. As described in the points above, the results presented do not support the statement in the abstract "CRISPR further indicated that rs17622517 is indeed a causal variant in this locus, and recapitulated the LPS-specific trans-eQTL signal"

RE: We have softened the phrasing of this sentence in the abstract to better reflect the results of the study: "*Genome editing by CRISPR had suggestive recapitulation of the LPS-specific trans-eQTL signal and lent support for the rs17622517 locus being causal.*"

Minor comments:

1. Could the authors clarify if the "strongest trans-eGenes" referred to on page 7 are those with the most significant eGenes rather than necessarily those with the greatest effect size?

RE: We could not find a reference to strongest trans-eGenes on page 7, and perhaps the reviewer was referring to the line on page 3, "The alternative allele of the lead variant rs17622517 is associated with upregulation of IRF1 upon early immune stimulus, and upregulation of 11 out of 12 of its strongest trans-eGenes as well". We meant that these are the 12 eGenes with the lowest FDR and therefore to clarify, we rephrased this part of the sentence to "11 out of 12 of its most significant trans-eGenes".

2. Page 7: how were the tagging variants identified? Could this SNP be tagging a structural variant?

RE: The reviewer raises a good point. We have included more details about the LD tagging analysis in the text. Structural variants were not included in the genotyping or imputation of the genotyped data. To explore this possibility, we accessed all structural variants within 50,000 bp of the top SNP rs17622517 in the gnomAD v3.1

SV dataset (https://gnomad.broadinstitute.org/). Below please find a table with all the structural variants in this region. The variants are all very rare and seem unlikely to be tagged by rs17622517, which has a MAF of 0.057 in gnomAD. The most common DEL_5_62909 has an AF of 0.005, however is almost exclusively found in African Americans in the dataset and therefore is unlikely to be driving the eQTL association in this European population. We now mention this in the text.

Variant ID	Consequence	Class	Position	Size	Allele Frequency
INV_5_294	loss of function	inversion	114283102-148759757	34476655	0.000046
DEL_5_62904	intronic	deletion	132424322-132424608	286	0.000046
INS_5_42763	intronic	insertion	132438226	529	0.000092
INS_5_42765	intergenic	insertion	132455621	62	0.000046
INS_5_42766	intergenic	insertion	132461531	1579	0.000046
DEL_5_62907	intergenic	deletion	132475046-132475545	499	0.001062
DEL_5_62906	intergenic	deletion	132475046-132475385	339	0.000047
INS_5_42767	intergenic	insertion	132483170	69	0.000046
DUP_5_16297	intergenic	duplication	132487275-132487357	82	0.000092
DEL_5_62908	intergenic	deletion	132491154-132499972	8818	0.000046
INS_5_42768	intergenic	insertion	132503837	1240	0.000046
INS_5_42769	intergenic	insertion	132509906	281	0.000784
DEL_5_62909	intergenic	deletion	132513084-132513652	568	0.005072

3. Beyond highlighting that the rs17622517 has a substantial number of trans-eGenes, Figure 1a is initially hard to interpret further. It may be more helpful to plot a histogram of how many cis SNPs have 1 trans-eGene, 2 trans-eGenes etc to highlight the rs17622517 locus but also give a better idea of how many eGenes are detected for the other SNPs.

RE: We agree that the information in fig 1a could be better represented. Therefore, we have taken the reviewer's suggestion to replace it with a histogram of the number of eGenes per SNP (right).

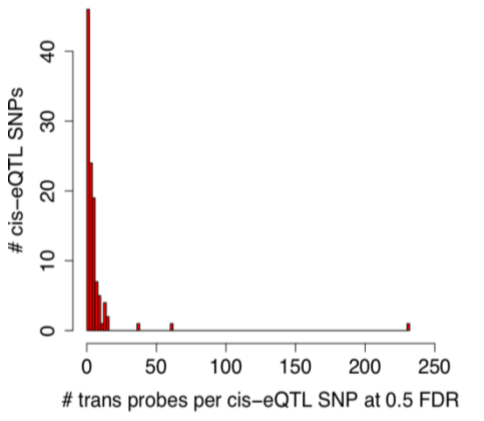

4. It would be helpful to include the p value and effect size of the cis eQTLs in figure 1b.

RE: Thank you, these have been added.

5. What does the shading represent on figure 1e?

RE: Thank you for pointing out this missing information. The shading represents the intensity of the transcription factor peak from ENCODE. We have updated the legend accordingly:

"e) overlap of rs17622517 (blue line) with ENCODE transcription factor ChIP-seq peaks (grey) and motifs (green). Intensity of grey scale is proportional to the intensity of the ChIP-seq peak."

6. For supplementary figure 5 could the authors clarify in the figure legend what comparisons have been made for the differential expression due to enhancer and promoter silencing in? Is this compared to the same time point in controls?

RE: Thank you for pointing out this missing information. The differential expression analysis in Supplementary Figure 9 (formerly supplementary figure 5) is compared to the same time point in the controls. We have updated the legend accordingly:

"Supplementary Figure 9. Enhancer and promoter silencing effects for different LPS time points. Number of significantly differentially expressed genes in promoter (a) and enhancer (b) versus controls at the respective LPS condition."

7. Are the authors concerned that the differential expression lists aren't enriched for IRF1 targets? Is it something else driving the difference in expression e.g. off target CRISPR effects, or is this related to the time points chosen?

RE: While the trans-eGenes are enriched for IRF1 targets, we do not see this enrichment in the CRISPRi promoter or enhancer differential expression. Naturally, this is a signal that we would have preferred to see, but we are not overly concerned. This is because we do see some enrichment for immune-related pathways, such as NFKB signaling (regulated by IRF1), and the hundreds of differentially expressed genes are likely to include a large number of affected genes further downstream in the pathway that are not directly regulated by IRF1 binding. It is conceivable that the pathway effect of the trans-eQTL SNP in primary monocytes could be more subtle and allow us to see mostly direct IRF1 targets, compared to the stronger CRISPRi perturbation in a cell line system that may allow detection of a more widespread pathway disruption.

Regarding the time points, we did test different time points in a pilot study and selected 6 hours because it had the highest expression of the most IRF1 trans-eGenes (Supplementary Fig 6), which are also enriched for IRF1 targets.

8. Fig 4c is cited in the text before fig 4a. Fig 4d is cited in the text before fig 4b.

RE: Fixed. Thank you.

Reviewer #2:

In « An autoimmune disease risk variant has a trans master regulatory effect mediated by IRF1 under immune stimulation », authors Margot Brandt et al. explore the trans-effect of genetic variation on the transcriptional response of primary monocyte upon LPS stimulation using a data set published previously in Kim-Hellmuth et al. (2017). They identified a risk variant for autoimmune disease, rs17622517, as a master regulator of the innate immune response upon late LPS stimulus through the regulation of IRF1 gene expression. To validate the role of this particular variant in such regulation, they performed CRISPR-based experiments in order to provide functional evidence of its causative effect.

Overall, the results presented by the authors are of interest since only few studies have attempted to develop an in vitro CRISPR-based approach in cellular models to characterize the functional effect of immune-responsive regulatory variants. However, I have major concerns about the conclusions highlighted by the authors.

The experimental work presented here suggest a trans-effect of the rs17622517 locus regulating immune response genes following LPS stimulation, but it is not sufficient to conclude that the effect is modulated by IRF1. Indeed, the authors did not show any direct evidence that the rs17622517 locus is an enhancer of IRF1 expression since the inhibition of this locus by CRISPRi has no effect on IRF1 expression (Wilcoxon p=0.8), as opposed to the inhibition of IRF1 promoter which exhibited a significant down-regulation of IRF1 (Wilcoxon p=7.5x10-7). The same remark can be made for the CRISPR/Cas9 genome editing experiments where the different deletions introduced in the rs17622517 locus have again no effect on IRF1 expression. In addition, the lack of power that they highlighted several times in the article, as well as the relaxed FDR thresholds that they use for the analysis indicate some weakness of the study and highlight the need to be replicated more deeply in order to have confident results. For all these reasons, the authors should be more cautious when claiming that the trans-effect of the rs17622517 locus is modulated by IRF1. The conclusions, as well as the title of this article, should be modified accordingly.

RE: We have performed additional experiments to address this concern, also raised by Reviewer 1. RE: We have performed additional experiments to address this concern and also revised the text. We performed CRISPRi silencing of the promoter and enhancer in the THP1 monocyte-like cell line, and showed that the enhancer silencing indeed affects IRF1 expression with upregulation without LPS stimulus and suggestive downregulation at 90 mins. These results are included in Fig 2b and shown below. This result provides bona fide evidence that the locus is an enhancer of *IRF1*.

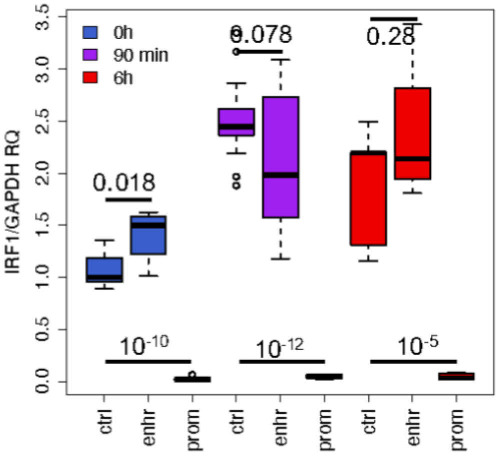

Furthermore, we now show that this variant is not a cis-eQTL for any other gene in our monocyte data (Supplementary Figure 2, copied below). We highlight that our claim of causality of IRF1 is not reliant on the CRISPR experiments alone; the cis/trans-eQTL discovery is also an important line of evidence.

Altogether, we feel that our combined eQTL and experimental results strongly support the mediating role of IRF1. There are multiple lines of evidence: 1) the two independent cis-eQTL effects on IRF1 (and no other genes in cis) with concordant trans-eQTL signals, 2) downregulation of IRF1 after silencing the enhancer locus in THP1 cells, 3) similar genome-wide expression effect of enhancer and promoter silencing as well as genomic perturbation of the locus.

We have, however, softened the claims across the manuscript to be transparent about the degree of evidence provided by the data presented in this work.

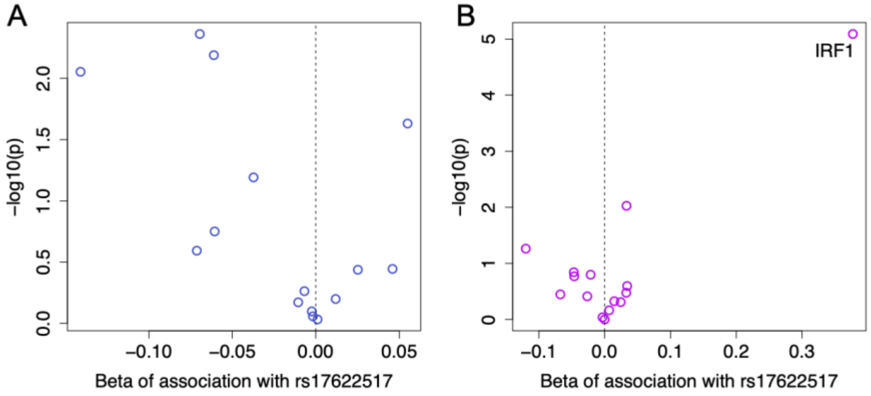

Moreover, the cellular model of HEK293-TLR4 cell line used here does not seem to be well suited to study IRF1 pathway. Although this cellular system is responsive to LPS stimulus, there is no enrichment of IRF1 target genes among differentially expressed genes upon

stimulation, as opposed to the results obtained in the primary monocytes. A comparison of the transcriptional response upon stimulation between monocytes and HEK293-TLR4 cell line would be informative (Venn diagram to see the proportion of genes expressed in both cell-types, correlation of the transcriptional response for genes commonly expressed, GO enrichment for these genes…). More particularly, are the 12 trans-eGenes the most strongly associated at FDR=0.05 in monocytes expressed in HEK293 – TLR4 cells? If so, is there a correlation in their expression?

RE: The reviewer raises a good point, and we have added data to the manuscript to further demonstrate that HEK-hTLR4 indeed recapitulates immune response in monocytes. While the entire IRF1 pathway is unlikely to be identical across cell types, we would like to note that the very strong gene expression effect of the CRISPi silencing of IRF1 promoter in HEK-hTLR4 shows that this gene has a functional impact in this cell line. Furthermore, the new THP1 experiments show that the enhancer locus affects IRF1 expression.

The analyses suggested by the reviewer are outlined below and now included in Supplementary Figure 5d-f, copied below. First, we compared the total expressed genes in monocytes and HEK-hTLR4 cells and found that the majority are expressed in both (Supplementary Figure 5d). Next, to analyze the similarity of immune response between HEK-hTLR4 cells and primary monocytes, we took the log2 fold change (log2FC) for the top 100 differentially expressed mutually expressed genes (by absolute value of fold change) in the HEK-hTLR4 cells and correlated with the log2FC in monocytes and found that they are well correlated (Supplementary Figure 5e). We also took the top 100 DE monocyte genes and performed the same correlation (Supplementary Figure 5f), which also shows a correlation, albeit more modest.

We were not sure what the reviewer meant by analysis of correlation in the expression of the 12 trans-eGenes. Seven of the 12 trans-egenes are expressed in the HEK-TLR4 cell line, defined as an average read count of greater than 5 across conditions.

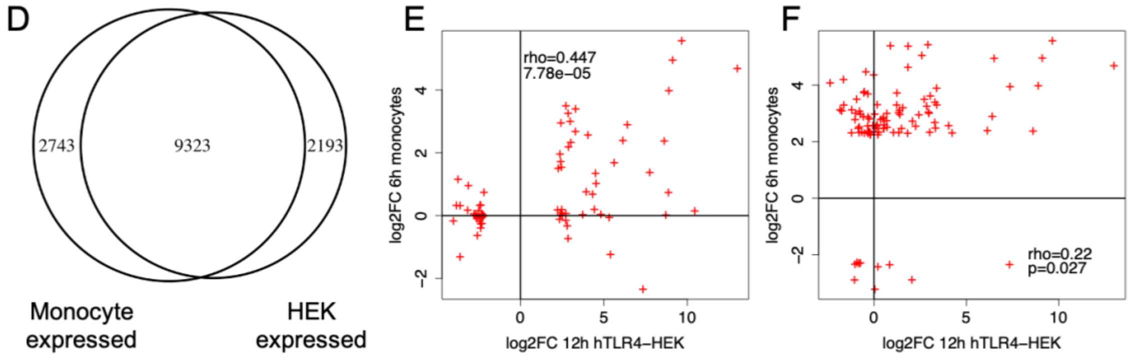

The CRISPR/Cas9 gene editing design used by the authors did not allow the discrimination of the trans-effect at the nucleotide resolution since they introduced deletions surrounding the rs17622517 locus and did not perform SNP editing. They delimited a DNA sequence where a regulatory element may be present and any nucleotide modification within this sequence, including rs17622517, may have a trans-effect. A comment on this issue in the article would be appreciable.

RE: We agree with the reviewer. In response to this point and a comment from reviewer 1, we have expanded on this in the discussion, adding the following text:

"While editing the exact variant into a cell line may reflect directionally the same genetic effect as seen in the population, a deletion of a locus could feasibly have an effect in the same direction, opposite direction or not at all. This depends upon the specific mechanism of the genetic effect. For example, if a variant increases the affinity of a DNA binding protein by modifying its binding motif, that effect may not be replicated by a simple deletion of the variant locus. However, a functional impact of indels does provide evidence that the specific SNP locus has a regulatory effect rather than being a nonfunctional LD proxy of another causal variant."

In addition, the results regarding the analysis of Het and Hom clones are not convincing because there is no down-regulation of IRF1 expression depending on the genotype classes, as expected. The trans-effect with differential expression in Het and Hom clones showed also an opposing direction of the correlations, which is counter-intuitive, and the variability within genotype classes seems very high. The authors used ranges of NHEJ rate to determine the different genotype classes (NHEJ<10% for WT, NHEJ=20-70% for Het and NHEJ>90% for Hom). Did they choose these ranges arbitrary or this decision was supported by biological/statistical arguments? Moreover, how did the authors estimate the number of clones to test per genotype class? What was the transcriptional variation among clones within each class? Given the moderate effect size of rs17622517 locus and knowing the transcriptional variability among clones within each class, they should calculate the statistical power to detect a biological effect using 13 clones.

RE: We chose the thresholds of 10%, 20-70% and 90% because there was noise in the estimation of NHEJ due to sequencing errors creating misalignment in the genotyping analysis. After using these thresholds to narrow down clones, we further inspected the BAM files manually in IGV to ensure that the ratios made sense in light of there being three alleles in each clone.

We don't have a great estimate of the size of the trans effects we would expect to see in RNA-seq, because the monocyte cells were characterized with microarray which is known to have a smaller dynamic range than RNA-seq and the values are not directly interpretable as counts. However, to answer the question on power, we did a power analysis using the R package "RnaSeqSampleSize" where for each of the 0.5 FDR trans-eGenes, we calculated the effect size that would be needed to detect with the number of clones we obtained and a power of at least 0.8. The results are in

the histogram below, demonstrating that although some genes would require a FC that is unlikely for this trans- effect, a large portion (59%) of genes would need a fold change between 1 and 1.5.

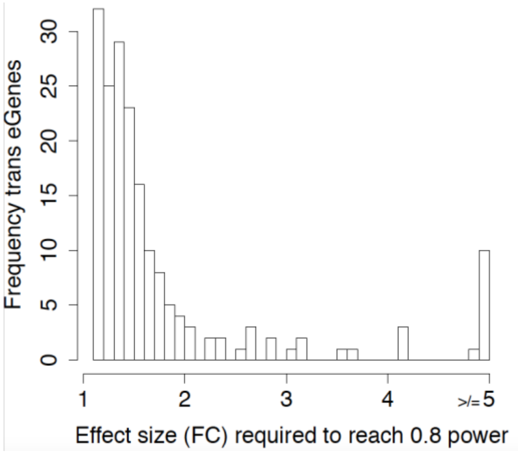

My general comment on this article is that the interpretation of the data should be more rigorous:

Depending on the biological hypothesis that the authors want to highlight, they considered different FDR thresholds, from 5% FDR up to 50% FDR. They should explain on which criteria they selected these thresholds and what are the consequences in terms of data significance. It reveals the clear lack of statistical power in their analyses, which results in a doubt about the robustness of the genetic associations.

RE: In our experience, is not uncommon in genomics to vary FDR thresholds to capture genome-wide signals at different levels of sensitivity and specificity. However, we use 5% FDR consistently with only two exceptions: 1) for *trans*-eGene discovery we cast a wider net by using 50% FDR, also because we make no specific statements about any individual genes and treat them as a group, 2) in the analysis of correlation of differential expression between CRISPR genotypes and CRISPRi, we use 25% FDR. We have now added this result at 5% FDR (Supplementary Figure 12e&f).

In the figure Fig. 1a, the authors reported that the rs17622517 locus presented "substantially" a larger number of trans-eGenes with respect to other loci at a FDR=0.5. First they should compare groups using a FDR of 0.05 and second test if the difference is statistically significant. In general, the authors should avoid the terms "substantially" or "suggestive" when no statistical support is provided.

RE: In the text, we had also reported the number of trans-eGenes at 5% FDR, and this locus stands out (12 eGenes compared to 4 for the next highest at 5% FDR, 232 compared to 62 next highest at 50% FDR). We now show this as a new supplementary figure 1 (figure 1a was similarly revised based on feedback from reviewer 1). We have

revised the sentence to: "One locus stood out by being associated with a larger number of trans-eGenes than any other: 12 at 5% FDR and 232 at 50% FDR".

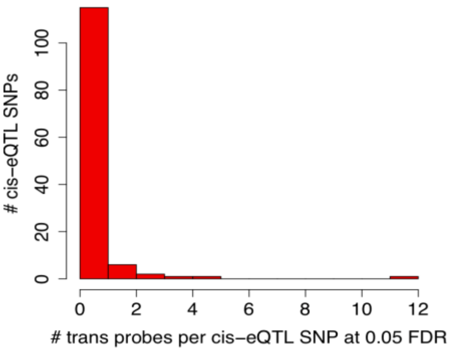

The authors mention that the lead variant rs17622517 had no LD-tagging variants but no information on the processing of the genotyping data has been reported in the article. What is the total number of SNPs considered in the analyses? Has SNP imputation been performed? Has the genetic sub-structure of the population been accounted for in the eQTL analysis? Has a fine mapping of the region been performed to make sure that rs17622517 was the best SNP associated with the disease, as well as with gene expression phenotypes?

RE: We have included additional details addressing these questions in the text, and following recommendations by Reviewer 1, performed new analysis of structural variants in the locus. Briefly, all the donors of the eQTL data were of German descent. Genotyping was performed on Illumina's HumanOmniExpress BeadChips comprising 730,525 SNPs, 579,090 of which passed QC and were used for analysis. Genotypes were phased with SHAPEIT2 and imputed with IMPUTE2 in 5 Mb chunks against the 1000 genomes phase 1 v3 reference panel. Fine-mapping of the GWAS locus was done in Ellinghaus et al 2016 and de Lange et al 2017.

By comparing the LD pattern of the top SNP (Figure 1D, first plot below) with that of the second top SNP (New Supplementary Figure 4a, second plot below), you can see that the architecture of the cis-eQTL locus is that the top p value SNP is not in high LD with surrounding variants, but the second top SNP is, suggesting they are actually tagging two independent eQTL at this locus, as we discussed in the text.

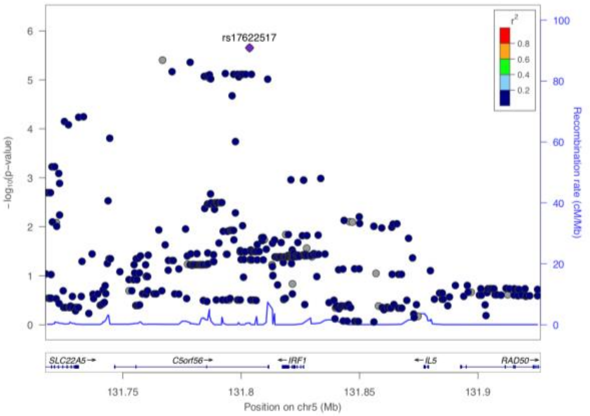

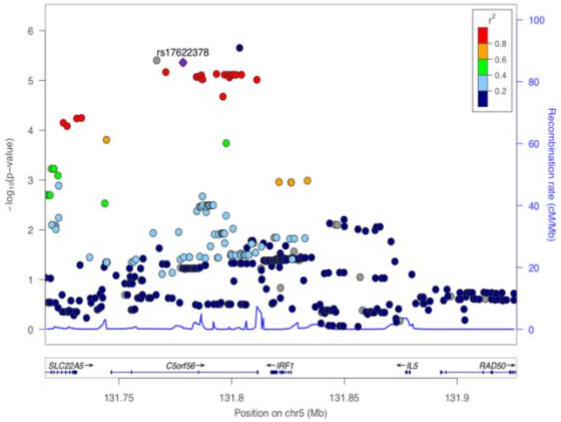

Several replicates have been performed in this study but no analysis of the replicates have been reported by the authors in the article, knowing that such analysis is essential for the validation of the experimental settings. Please provide measures of technical variability and show that the biological effect that you expect is higher. Regarding the two replicates that were done for each of the 13 clones, the reads from the replicates were combined. Did the authors quantify the technical noise between replicates? Did they perform some quality checks to ensure that the sequencing data from the two replicates could be merged (correlations, variation between replicates…).

RE: We have now included in Supplementary Figure 11 analyses that demonstrate concordance of replicates and robustness of the data. We had indeed checked with PCA that the technical replicates were concordant before merging them, and notably all samples were included in the replicate experiments in order to avoid confounding by condition or genotype. You can see in the first figure below (new Supplementary Figure 11a) that PC1 does separate the samples by experiment, while PC2 separates samples by LPS treatment. This is not surprising for a set of samples from two balanced batches. However, PCA of the control samples shows that clones tend to cluster together, confirming that the clone-specific effects were preserved in the two experiments (Supplementary figure 11b).

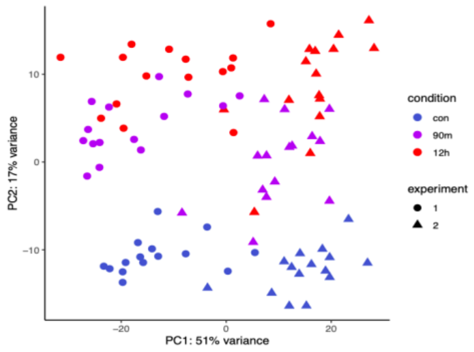

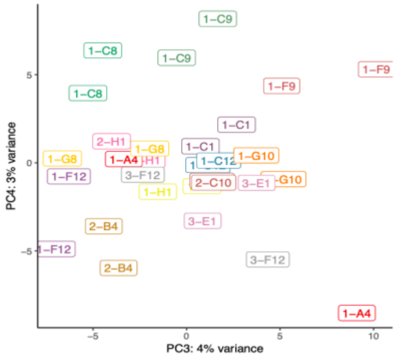

Regarding in the analyses ran in the Figure 3 d-e, the authors used the set of genes that were significantly differentially expressed in heterozygous lines compared to homozygote lines because the effect of the genetic mutations was best captured with respect to the other genotype comparisons. Please provide the biological sense of comparing the fold change of these genes with the fold change obtained in the CRISPRi promoter vs control or in the CRISPRi enhancer vs control. Such comparisons should be performed with the list of genes captured in Het vs WT or Hom vs WT, instead of Het vs Hom.

RE: We have added the comparisons of the other genotype groups (new Supplementary Figure 12a-d, copied below). There is a mild negative correlation in WT vs Het, but the others are nonsignificant. We have clarified this analysis and its interpretation in this section as follows, also softening the conclusions:

"Testing differential expression between genotypes specifically at 12h, we saw the highest number of differentially expressed genes in comparison of homozygous and heterozygous clones (762 differentially expressed genes at 25% FDR, < 10 for other genotype comparisons). This discordance in numbers may be due to different effects of indels of slightly varying size and the cell lines potentially having other on-target or off-target mutations not detected by our genotyping assays (Supplementary Figure 10). Nevertheless, hypothesizing that these 762 genes best reflect the impact of genomic disruption in this locus, we compared the gene expression response to that in the CRISPRi silencing experiment. We discovered a significant correlation of the fold changes with those from both promoter (rho=0.21, p=2.52e-8) and enhancer (rho=0.22, p=4.31e-9) silencing (Fig. 3d and e). Correlations for the top 100 FC genes in other CRISPR genotype comparisons are shown in Supplementary Figure 12. These results suggests that disruption of genomic sequence at the rs17622517 locus can have functional effects that are partially similar to downregulation of IRF1, even though we are underpowered to detect effects on individual genes in cis and largely also in trans."

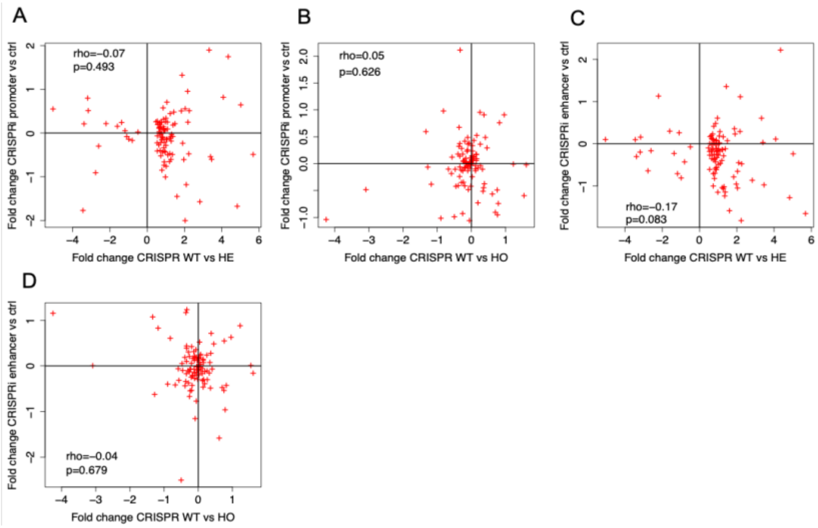

Minor comments:

When multiple expression probes measure the same gene, what was the correlation between probes for the trans-eGenes?

RE: To answer this question, we took all the trans-egenes (FDR < 0.5) with more than one trans probe (n=19) and correlated the first and the second probe eQTL effect size (beta) to see whether the variant had the same effect on the different probes. We found the probes to be highly correlated (rho = 0.81, p = 2.5e-5), plotted here and now mentioned in methods:

"Effect sizes from probes for the same gene were highly correlated (rho = 0.81, p = 2.5e-5)"

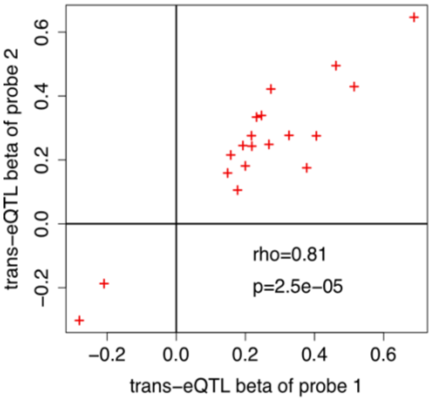

Is the background gene set used in the enrichment analysis for IRF1 target genes based on a pool of genes expressed in all conditions or only on gene expressed in the condition of interest?

RE: For the background gene set we used genes expressed in monocytes (n = 15,174), minus the IRF1 target genes. These are derived from the list of probes which were used in the cis-eQTL analysis, including probes which had a p of detection < 0.01 in at least 10 samples across all conditions. We clarified this in the methods.

Was the expression data corrected for technical variability?

RE: We are not entirely sure which expression data the reviewer is referring to. In the trans-eQTL analysis we did not use PEER or other techniques that are commonly used to remove technical and other nuisance variation from cis-eQTL analysis, since they can lead to both false positives and negatives in trans-eQTL analysis. The stimulation and RNA-seq samples were processed and sequenced in one batch for each experiment, except for the CRISPR clone experiment which was repeated for all samples and the two replicate experiments were combined.

Please add in the supplementary tables 3 and 5 the name of the genes using HUGO nomenclature.

RE: Thank you for pointing out this oversight. These have been added.

In Supplementary Figure 6B, the orange line for gDNA should be replaced by the genomic sequence of the region.

RE: Thank you. This has been added.

Regarding the clones edited by CRISPR/Cas9, it would be appreciable to have their ID on the corresponding figures.

RE: We tried to do this for Fig. 3C, but the figure became very messy.
