# Supplementary material for: An autoimmune disease risk variant: A trans master regulatory effect mediated by IRF1 under immune stimulation?
Source: PLoS Genet. 2021 Jul 27;17(7):e1009684. doi: 10.1371/journal.pgen.1009684 (PMC8345867; doi:10.1371/journal.pgen.1009684)
Supplement: Attachment — Submitted filename: IRF1_response_R2.docx [file pgen.1009684.s022.docx]

***Response to the editor and reviewer comments***

As suggested by the editor, we have changed the title to "*An autoimmune disease risk variant: a trans master regulatory effect mediated by IRF1 under immune stimulation?*". Furthermore, we have revised the text to add acknowledgement of limitations of our approach, especially in Discussion, as suggested by the reviewers.

Reviewer #2:

In the revised version of “An autoimmune disease risk variant has a trans master regulatory effect mediated by IRF1 under immune stimulation”, the authors Margot Brandt et al. have significantly improved the quality of the manuscript by bringing new experimental data (i.e. generation of THP-1 CRISPRi cell lines and RT-PCR experiment to measure IFR1 gene expression) in order to support the trans-effect of rs17622517 on immune response following LPS activation, via the regulation of IRF1. They have also added supplementary analyses (i.e. comparison of LPS response in HEK293-hTLR4 cells versus monocytes) and technical information that allow a better understanding of the article.

The association between rs17622517 and IRF1 expression remains suggestive, preventing any clear demonstration of a trans-effect of rs17622517 following LPS activation, even in THP-1 cells, as shown in the new figure 2b. Regarding this new figure, enhancer silencing leads to significant upregulation of IRF1 at 0h LPS (t-test p= 0.018). Since we do not expect an effect of the enhancer at baseline on the basis of the eQTL mapping performed in primary monocytes (Figure 1b), please comment on your results. In addition, the authors used a t-test to show a suggestive down-regulation of IRF1 at 90 min LPS (t-test p=0.078), but nothing was specified about the normality of the distribution. According to the low number of samples, I think a Wilcoxon rank-sum test would be more appropriate. I suggest that the authors re-calculate the p-values accordingly. Finally, to appreciate the variation of IRF1 expression among THP-1 clones, the box plot should show the median value with the interquartile range and individual values should be added to the figure.

In general, the authors have made a lot of efforts to discuss the lack of power in their experimental settings preventing them to clearly demonstrate the causal role of rs17622517 in the inter-individual response to LPS stimuli via regulation of IRF1, and have softened their claims accordingly.

**RE: We thank the reviewer for encouraging comments and good suggestions. We have changes the statistical test to Wilcoxon rank-sum test, and revised to figure to show individual data points. This does not change conclusions of the analysis, and the previously significant results remain significant.**

Reviewer #3:

I am commenting on the revised version of this manuscript, not having given feedback on the original. In general, the authors have made substantial revisions in light of the comments on the original manuscript. They show several lines of evidence which, whilst individually weak, suggest that a variant near IRF1 is associated with IRF1 expression levels in early macrophage LPS response, and with differences of IRF1 targets in trans later in the same response. The CRISPRi data suggest this region, which harbors a variant associated to IBD, is an enhancer, as there are marginal effects on LPS-induced IRF1.

This work is a good cautionary tale in how hard it can be to unravel trans-eQTLs - both temporally and in terms of effect size. The authors have to compile multiple strands of individually incomplete evidence to arrive at their current narrative. I have a couple of things for them to consider:

- There is no co-localization analysis between the IBD GWAS data and at least the IRF1 cis-eQTL data, which might indicate a shared effect. In the title and discussion, the authors imply that this is likely a disease-relevant mechanism, but some acknowledgement of the limited resolution of fine-mapping should be made if no sharing can be shown.

**RE: We have added text to the discussion to acknowledge that the complex genetic architecture of this locus makes standard colocalization analysis difficult.**

- IRF1 is expressed in a wide variety of immune cell types. The possibility that this mechanism, if relevant to disease, may be acting in some other cell type deserves some acknowledgment.

**RE: We already had a sentence about this in the Discussion, but we have now highlighted this point in more detail.**

- There is no discussion or data on whether this trans-eQTL actually changes anything about macrophage response to LPS. If no cellular phenotype can be detected, why would this effect be likely to have a broader physiological effect like influencing disease risk? I would suggest this merits at least some discussion as a limitation on the interpretation of the data presented.

**RE: This is a major challenge for all GWAS follow-up studies. We have emphasized this point in the Discussion in further detail.**
